# The Crab Hole Mosquito Blues

**DOI:** 10.3201/eid1705.101412

**Published:** 2011-05

**Authors:** Karl M. Johnson, Douglas F. Antczak, William H. Dietz, David H. Martin, Thomas E. Walton

**Affiliations:** Author affiliations: Retired (K.M. Johnson, T.E. Walton);; Cornell University, Ithaca, New York, USA (D.F. Antczak);; Centers for Disease Control and Prevention, Atlanta, Georgia, USA (W.H. Dietz);; Louisiana State University Health Science Center, New Orleans, Louisiana, USA (D.H. Martin)

**Keywords:** viruses, Venezuelan equine encephalomyelitis virus, VEE, Deinocerites pseudes, Culex (Melanoconion) spp., arbovirus, VEE virus strain TC-83, attenuated vaccine, music, Another Dimension

## Abstract

Venezuelan equine encephalomyelitis (VEE) epizoodemics were reported at 6–10-year intervals in northern South America beginning in the 1920s. In 1937, epizootic VEE virus was isolated from infected horse brain and shown as distinct from the North American equine encephalomyelitis viruses. Subsequently, epizootic and sylvatic strains were isolated in distinct ecosystems; isolates were characterized serologically as epizootic subtype I, variants A/B and C; or sylvatic (enzootic) subtype I, variants D, E, and F, and subtypes II, III, and IV. In 1969, variant I-A/B virus was transported from a major outbreak in northern South America to the borders of El Salvador, Guatemala, and Honduras. This musical poem describes the history and ecology of VEE viruses and the epidemiology of an unprecedented 1969 movement of VEE viruses from South America to equids and humans in Central America from Costa Rica to Guatemala and Belize and in Mexico and the United States that continued until 1972.

## Crab Hole Mosquito^A^ Blues

### Written and performed by the MARU Health Angels Band^B^

**Refrain**: Mosquito’s in his^C^ crab hole, bidin’ his time,

Venezuela virus working up the line,

Boys in Beltsville^D^ heard the news,

Horses in Texas^E^ got the crab hole blues.

Down in Maracay back in ’36,

Kubes and Rios^F^ found a virus doin’ tricks,

Horses die, this one’s gotta be,

New cause of ’cephalitis, V-E-E.^G^

**Voice-over**: Horses, mules, and donkeys are all susceptible.

The years roll by, the virus makes a score,

In Vene, Colombo, and Ecuador,^H^

Comes rain to the desert instead of dew,

And, VEE burns the coast of Peru.^I^

In Trinidad, Panama, they say “Hey, Hey,”

We got this creature like every day,

His swampy home, you can always tell,

By finding some rats and the *Culex* (*Mel*).^J^”

**Voice-over**: Mosquito, that is.

Back at MARU, raggin’ the brains,

We found that the virus had different strains

Horses convulse with the southern kind,

Northern virus leaves them feelin’ fine.^K^”

**Refrain.**

’Cause virus hits man by aerosol,

Could be used to cripple us all,

In Maryland, they grew it in vats,

Tested it out in monkeys and rats.^L^

The other Team outside the fence,

Lookin’ for the answer that made some sense,

Got a strain from an old donkey,

Made a vaccine, TC-83.^M^

The offensive team tried to make some noise,

A cloud for the Commies, but not our boys,^N^

Did not work, that is why,

Nixon^O^ said ‘declassify’.

**Voice-over**: The Pentagon papers, volume 25, page 324, 1970… the New York Times, Los Angeles Times, the Washington Post, and the Christian Science Monitor….

Sleepy defense gets a Latin call,

New outbreak and it ain’t small,

Took the vaccine off the wall,

It really worked to save them all.^P^

**Refrain**

Down in Guatemala, year of ’69,

Horses started dying on the borderline,

Virus come down from Ecuador,

Nobody knows the reservoir.^Q^

**Voice-over**: But Mackenzie^R^ has an idea.

Virus hopped the mountains, got to the sea,^S^

Vaccine brought in and given out free,

Virus kept movin’, had to go,

All the way from here to Mexico.^T^

Cause everybody said, “Gotta stop that bug,

Gotta use the vaccine because there ain’t no drug,”

Beltsville brethren said “Gotta wait,”

Afraid that the vaccine was MF-8.^U^

**Voice-over**: That means reversion to virulence.

Results today are plain to see,

Story’s hot on the wire of Associated P,^V^

A million horses all over the West,

Are living proof the vaccine was best.^W^

**Refrain**

**Voice-over**: Addresses for reprints may be mailed to Karl M. Johnson.

A) The presentation is a chronologically, scientifically, and factually correct poetic and musical history of Venezuelan equine encephalomyelitis (VEE) viruses and epidemics through 1971. The music is a traditional US jug band song, a type of music—bluegrass—popular in rural areas of the eastern United States. Using a background of a guitar and everyday musical, rhythm, and percussion devices, such as whistling, blowing air across the mouth of a 1-gallon glass jug, scraping of a scrub brush on a metal washboard, humming harmonically into a kazoo, and the “hambone” (a rhythmic slapping of hands on arms, hands, and legs), the voices and lyrics convey a musical story to the audience.

The title reflects the discovery that *Deinocerites pseudes*, the crab hole mosquito, found along the Pacific coast of Central America was a competent vector of the epidemic and epizootic VEE virus that was causing disease and death in equids and humans at the time (*1*). The poetic dialogue was written and set to music in 1971 by scientists at the Middle America Research Unit (MARU), US Department of Health, Education, and Welfare (now Health and Human Services), National Institutes of Health, National Institute of Allergy and Infectious Diseases, US Public Health Service, and located in Ancon, Panama Canal Zone, during a major western hemispheric outbreak of VEE. MARU scientists had a >15-year history of VEE epidemiologic and virologic studies throughout Latin America to characterize the antigenic relationships between South American VEE virus strains of subtypes (I-A/B and I-C) that caused epizoodemics of equine and associated human disease and the Caribbean, Central American, Floridian, Mexican, Panamanian, and South American VEE virus strains of subtypes (I-D, I-E, I-F, II, III-A, III-B, and IV) that existed in sylvatic, enzootic cycles in the absence of equine disease but with occasional human infections and disease (*2*). In 1969, the transfer of equine virulent VEE virus from a raging epizoodemic in Colombia, Ecuador, and Peru to the frontier area of El Salvador, Guatemala, and Honduras precipitated a human and veterinary medical crisis in Central America, Mexico, and the United States that lasted until 1972 (*3*).

The Crab Hole Mosquito Blues was written as a scientific presentation for the international Workshop-Symposium on Venezuelan Encephalitis Virus sponsored by the Pan American Sanitary Bureau, Pan American Health Organization, World Health Organization, in Washington, DC, September 14–17, 1971. Although the lyrics of the Crab Hole Music Blues were not published in the Proceedings, other presentations from that meeting are documented (*4*).

B) Douglas F. Antczak, vocals, guitar, kazoo; William H. Dietz, vocals, jug, kazoo; Karl M. Johnson, vocals, kazoo; David H. Martin, kazoo, recording engineer; and Thomas E. Walton, washboard and scrub brush, hambone, kazoo, vocals.

C) Gender-specific error. Only female mosquitoes (and females of other hematophagous insects, e.g., culicoids, phlebotomids) take a blood meal, which is needed to provide protein necessary for ovulation; only female mosquitoes are infected with and transmit VEE viruses (and other mosquito-borne arboviruses). Male mosquitoes feed on plant source liquids and water.

D) Beltsville, Maryland, USA, the headquarters at that time of the regulatory officials in the US Department of Agriculture (USDA) responsible for the decision to recommend application of preventive vaccines to the Secretary of Agriculture in the face of an epizootic. Hesitation to vaccinate was predicated on lack of evidence that reversion of virulence of the attenuated vaccine virus could not occur, international trade considerations, international practices and agreements, and authorities and responsibilities delegated only to the Secretary of Agriculture.

E) From 1969, the epizoodemic moved southeast, eventually reaching northwestern Costa Rica in August 1970, and northward, eventually reaching Texas in late June 1971 (*4*).

F) The Venezuelan agricultural and veterinary research laboratories are located in Maracay, Aragua State, Venezuela. Kubes and Rios first isolated, identified, and named VEE virus, then sent the isolate to the United States for confirmation that the South American virus was antigenically distinct from the North American eastern and western equine encephalomyelitis viruses (*5**,**6*).

G) The antigenically related and clinically similar eastern and western equine encephalomyelitis viruses had been isolated and identified early in the 1930s in the United States (*7**,**8*).

H) Periodic outbreaks of VEE had occurred in Colombia, Ecuador, Peru, and Venezuela since at least the 1920s, with hundreds of thousands of equine illnesses and tens of thousands of deaths; equids are the primary virus amplifier hosts for human infections (*9**,**10*).

I) Throughout the history of VEE in northern South America, periodic epizoodemics in tropical dry and tropical thorn forests were often associated with unseasonably heavy rainfall and flooding during the normal dry seasons; during interepizootic periods, epizoodemic virus could not be isolated. The great Atacama Desert stretches along the Pacific coast from northern Chile and along coastal Peru nearly to the border with Ecuador; rare, but occasional, rainfall interrupts the barrenness of this parched, hostile environment permitting infrequent but noteworthy incursions of mosquitoes and epizoodemic VEE virus (*4*).

J) In contrast, in swampy or jungle areas (tropical wet forest) where a definable dry season does not occur normally in countries of Central America and eastern South America and in Panama, Mexico, the Florida Everglades, and several Caribbean islands, field studies by scientists at MARU (*11*), the Center for Disease Control (now Centers for Disease Control and Prevention, Atlanta, GA, USA) (*12*), the Trinidad Virus Research Laboratory (Port of Spain) (*13*), Rockefeller Foundation Laboratory (Belem, Brazil) (*14*), and the Gorgas Memorial Laboratory (Panama City, Panama) (*15*) had demonstrated presence of antigenically related VEE virus strains; resident equids in these sylvatic foci had antibody without signs of disease, but incursions by humans into these sylvatic or endemic areas often resulted in infections and disease. Sylvatic cycles were found in swampy areas in which floating species of water lettuce, *Pistia stratiotes*, provided appropriate habitat for mosquito species of the subgenus *Culex* (*Melanoconion*), the vectors of sylvatic subtypes and variants of VEE virus. The epidemiologic cycle involves sylvatic virus transmission by species of terrestrial rodents and possibly birds and arboreal rodents (*4*).

K) Sylvatic virus subtypes were of low or no virulence to experimentally infected equids. The virulence of epizootic subtypes was high, with fatality rates to >90% of infected equids (*16*).

L) Historically, VEE virus has been a pathogen studied for aerosol release as a potential biologic weapon. In the United States at the Army Research and Development Command, (now the Army Medical Research Institute for Infectious Diseases, USAMRIID), Fort Detrick, MD, the former Soviet Union and perhaps, elsewhere, VEE virus was studied as a possible offensive weapon. (For more information about the history of the US biologic warfare program and Fort Detrick, go to the following websites: www.detrick.army.mil/cutting_edge/index.cfm, www7.nationalacademies.org/archives/cbw.html, and www.bordeninstitute.army.mil/published.html.)

M) In the defensive research programs at USAMRIID, an equine-virulent isolate from a donkey was serially passed in fetal guinea pig heart cell cultures to produce an attenuated vaccine, strain TC-83, for use in at-risk laboratory and military personnel (*4*). Attenuated strain TC-83 was derived from the Trinidad donkey number 1 isolate from a diseased donkey in that country during a 1940s epizootic that spilled over to Trinidad and Tobago from mainland Venezuela (*17*).

N) “Commies” [communists] reflected Cold War–era concerns about military personnel of the former Union of Soviet Socialist Republics and their allies. Aerosol releases of biological, chemical, and radioactive weapons are notoriously difficult to control, leading to use of vaccine, if available, and other, more cumbersome measures to protect military personnel.

O) Recognizing the difficulties in controlling and using biological weapons, unauthorized release of classified documents, and the moral outrage of US citizens and world public opinion against biological weapons, President Richard M. Nixon cancelled the offensive biological weapons development programs.

P) Beginning in 1967, a major epizoodemic of VEE occurred across northern South America (*4*). Requests to the US State Department and US military authorities resulted in release of attenuated VEE virus vaccine strain TC-83 for emergency use in equids to stop equine disease and interrupt human infections. Vaccine was effective, but the silent epizoodemic tongue of virus transmission repeatedly had moved ahead of the vaccination teams through populations of susceptible hosts and competent vectors.

Q) In an unprecedented biomedical event in the history of VEE, the epizoodemic VEE virus subtype was transported from northern South America to Central America (*3*). Empirical and circumstantial evidence, such as discovery by scientific investigators of empty vials of VEE virus vaccine labeled by a manufacturer in South America, suggested that a formalin-inactivated VEE virus vaccine was imported to vaccinate valuable horses at breeding farms in Guatemala by worried ranch owners (K.M. Johnson, unpub. data). VEE virus, like poliovirus and other viruses, is notoriously difficult to inactivate. Safety tests of such inactivated VEE virus vaccines in laboratory systems, e.g., cell cultures and laboratory animals, are exquisitely less sensitive than susceptible equids to residual active virus. Non–inactivated virus has been postulated to replicate in equids to high titers and to be infectious for the local populations of competent and capable mosquito vectors.

The reservoir of epizoodemic VEE virus during interepizootic periods is unknown, but studies during the 1990s and 2000s suggest an enhanced virulence of certain isolates of sylvatic virus subtypes and strains for equids, which occasionally are replicated to high titers under undetermined favorable conditions, with subsequent selection of an epizoodemic clone from a mixed virus population that causes clinical VEE and infects mosquitoes.

R) An internationally recognized clinical and field research expert on zoonotic diseases, Ronald B. Mackenzie was a visionary and astute medical scientist with The Rockefeller Foundation in Cali, Colombia.

S) VEE virus was transported by mosquitoes and possibly through transportation of asymptomatic but infected horses from the disease or danger zones to unaffected zones where susceptible equids were subsequently infected. Disease was documented along the Pacific and Caribbean coasts in the tropical dry and thorn forest environments that have been the traditional cattle-raising areas of Central and South America and in Mexico, where tropical wet forest and swampy environments that support sylvatic VEE viruses are irregularly located and noncontiguous or do not occur.

T) Because of the lack of understanding of the epidemiologic cycle and virus-vector incubation requirements, the epizoodemic wave of infected mosquitoes and equids incubating the virus had not been anticipated to precede vaccination teams routinely into new areas of susceptible equids by 2–3 weeks. The disease moved to the southeast along the Pacific coast to northwestern Costa Rica, where the advance finally was stopped, protecting Panama, probably because of a combination of vaccination, presence of larger sylvatic foci in which larger numbers of resident equids were already immune, and a belt of lowland and montane rain forests where there are fewer cattle and horses and that stretches along the Pacific coast of southwestern Costa Rica to northwestern Panama; antibody to sylvatic virus strains provides cross-protection against epizoodemic virus strains. The virus crossed the Isthmus of Tehuantepec in Mexico and moved up both the Caribbean and Pacific coasts, finally reaching Texas in late June 1971 (detection of VEE in Texas before July 4 was predicted months earlier by K.M. Johnson). Despite thorough vaccination and aerial application of insecticides, some disease activity persisted in Mexico until 1972. The last activity from the epizootic in the Western Hemisphere occurred in 1973.

U) MF-8 is an isolate of the epizoodemic VEE virus subtype I-A/B from Honduras isolated by Miguel Figueroa, a Honduran scientist working at MARU. Despite proven efficacy and safety of the attenuated VEE virus vaccine in South and Central America and Mexico, USDA authorities delayed application of the vaccine until the first cases were diagnosed in Texas. Official USDA emergency response policies and regulations at that time did not include the option to use vaccines to interdict threats of foreign animal diseases in the absence of documented disease within the United States (application of vaccines in the USDA Emergency Response Plan to incursions of foreign animal diseases was authorized in 2000). Because of bilateral and international agreements and policies regarding emergency disease responses, the adverse impact of applying foreign animal disease vaccines on the exportation of US livestock and agricultural products internationally and the politically charged decision to change existing policy, an authority delegated only to the US Secretary of Agriculture, vaccine was acquired and stockpiled along the Mexico–US border but could not be applied until VEE was diagnosed.

V) Associated P = the Associated Press news agency.

W) Attenuated VEE virus vaccine was safe, effective, and stable, and reversion to virulence did not occur (*18**,**19*). A potentially catastrophic disaster was marginalized and ultimately stopped in the Western Hemisphere by application of vaccine and other control techniques. Hundreds of thousands of equids and thousands of humans were saved by the emergency responses of veterinary and medical officials in every country from Colombia-Ecuador-Peru-Venezuela to Mexico and the United States; among the 13 at-risk nations, only Panama (and, in addition the Caribbean islands and other countries of South America) was spared from this crisis.

The successful application to veterinary use of a vaccine developed by the US military as a defensive tool for use in troops and at-risk laboratory personnel was an unforeseen and unanticipated benefit of the US Department of Defense research program. President Franklin D. Roosevelt authorized the US Army through a civilian agency to develop the US biological warfare program with offensive and defensive objectives in 1942. Laboratories and pilot plants were constructed at Camp (later Fort) Detrick, Maryland; the Special Procedures program from which the Special Immunizations Program evolved was one of the earliest operations to open. The Special Immunizations Program is responsible for the investigational vaccines, including strain TC-83, which were developed and are used under the Investigational New Drug authority and guidelines of the Food and Drug Administration. Seed stock of strain TC-83 virus was made available to the biologics industries of Western Hemisphere countries. Strain TC-83 and other next-generation iterations of the original vaccine, including an inactivated strain TC-83 product (C-84), are used or available in many countries of the Western Hemisphere.
